# High-throughput sequencing analysis of intestinal flora changes in ESRD and CKD patients

**DOI:** 10.1186/s12882-019-1668-4

**Published:** 2020-01-13

**Authors:** Jianguang Hu, Xiaoshi Zhong, Jing Yan, Daoyuan Zhou, Danping Qin, Xiao Xiao, Yuanyuan Zheng, Yan Liu

**Affiliations:** 1 Department of Nephrology, Guangzhou Red Cross Hospital, Medical College, Jinan University, Guangzhou, 510220 China; 2 Guangzhou Institute of Disease-Oriented Nutritional Research, Guangzhou, 510220 China

**Keywords:** High-throughput sequencing, Intestinal flora, Hemodialysis, End-stage renal disease, Chronic kidney disease

## Abstract

**Background:**

Chronic kidney disease (CKD) disease affects gut flora by causing dysbiosis and lead to systemic inflammatory conditions. Here, we provide intestinal flora changes of CKD patients undertook different hemodialysis therapy.

**Methods:**

From 2017 to 2019, a total of 166 patients from Guangzhou Red Cross Hospital were recruited and divided into four groups with 17 cases in healthy control group, 47 cases in CKD non-dialysis group, 49 cases in HD group, and 53 cases in PD group. Intestinal flora genome 16S rDNA sequencing and further bio-informatic analysis were performed.

**Results:**

Decreased diversity and altered communities of intestinal flora in PD patients, in which microbial diversity was positive correlated with the albumin level were observed. A total of 20 intestinal flora phyla were detected in 166 fecal samples, divided into 3 dominant intestinal types including Bacteroides-dominant gut type, Firmicutes-dominant type and Proteobacteria-dominant gut type. Further analyses found 198 genera, the abundance of 86 genera were significantly different. Butyrate-producing taxa as *Faecalibacterium* in genera level and *Bifidobacteriaceae* and *Prevotellaceae* in family level were dominant genus in CT, CKD, and HD groups, while urease containing-, indole- and p-cresol-forming taxa as *Escherichia* in genera and *Enterobacteriaceae*, *Enterococcaceae* in family level was dominated genus in PD group. Number of differential expressed genes in KEGG enrichment pathways were significantly different in PD group in carbohydrate metabolism, amino acid metabolism, energy metabolism, translation, and membrane transport.

**Conclusion:**

Our results suggest peritoneal dialysis therapy could result in reduced diversity and altered microbial communities, with reduced probiotic butyrate-producing taxa and increased urease containing-, indole- and p-cresol-forming taxa. The disordered intestinal flora can seriously affect the nutrition level in CKD patients with PD therapy.

## Background

The human microbiome is comprised of about 100 trillion microbial cells and their encoded genes [1]. The composition of the gut microbiome varies among individuals and remains stable under homeostasis [2, 3]. The most abundant bacterial phyla in a healthy human gut are Bacteroides, Firmicutes, Actinobacteria, and Proteobacteria [4]. Genetic and environmental factors, including disease, diet and antibiotic use, alter the type and abundance of the microbiome [2]. This alteration, also known as dysbiosis, causes individuals to become more susceptible to disease [5]. Hormones such as serotonin, dopamine, and norepinephrine, and microbial metabolites including p-cresol sulfate, decyloxysulfate, trimethylamine N-oxide (TMAO), and short-chain fatty acids (SCFAs) secreted by gut microbiota, can influence various bodily functions [6, 7]. Kidney disease, obesity, metabolic syndrome, cancer, and cirrhosis were reported to be associated with changed endogenous flora [8–10]. So far, thorough investigations into gut-microbial-metabolite relationships under disease progression remain unclear.

Uncontrolled metabolic disorders including CKD, affect gut flora, promote intestinal permeability, cause dysbiosis, and can lead to systemic inflammatory conditions [11]. Reduced abundance of *Lactobacillaceae* and *Prevotellaceae* in CKD patients has been reported, while *Enterobacter* and *Enterococcus* were observed to be 100 times higher [12]. The secretion of uremic toxins is closely related to microbial changes in CKD patients [13]. Intestinal microbiota were associated with inflammatory status and renal function in end-stage renal disease (ESRD) patients in southern China, with a decreased proportion of bacteria, and altered intestinal flora from *Prevotella* to *Bacteroides* [14]. CKD animal models have shown excessive uremia can result in intestinal dysbiosis, intestinal barrier dysfunction, and bacterial translocation [15]. Intestinal bacterial changes were found in both dialysis and non-dialysis CKD patients. Further investigations indicated that the abundance of *Firmicutes* and *Actinobacteria* in peritoneal dialysis patients was reduced, and the abundance of Bacteroides in hemodialysis patients increased [16]. However, the influence of different renal replacement therapies on microbiota remains unclear.

In this study, we examined changes in the intestinal flora of CKD patients by comparing differences in abundance, diversity, and species composition between healthy humans, CKD non-dialysis patients, HD patients, and PD patients, providing evidence for personalized treatment for CKD patients.

## Methods

### Patient selection

From 2017 to 2019, patients from Guangzhou Red Cross Hospital were recruited and divided into four groups: healthy control group, CKD non-dialysis group, HD group, and PD group. The written informed consent was signed by all patients. This study was approved by ethics committee of the Guangzhou Red Cross Hospital [No. 2017–032-01/02]. The study adhered to the tenets of the Declaration of Helsinki and the Guidance on Sample Collection of Human Genetic Diseases by the Ministry of Public Health of China. The inclusion criteria for patients in the experiment were as follows: 1) receive normal diet; 2) capable of self-care; 3) no antibiotics, anti-tumor drugs, immunosuppressants, and glucocorticoids over the past 3 months. The exclusion criteria for patients included the following: 1) those who cannot eat or use intestinal and/or external nutrition interventions; 2) digestive diseases including gastrointestinal cancer, biliary tract inflammation, and inflammatory bowel disease; 3) metabolic diseases including obesity, diabetes, and systemic lupus erythematosus; 4) those who with local inflammation, systemic infections before treatment. Basic clinical information of all patients was recorded. Posterior feces samples were collected and stored at − 80 °C degrees for further analysis.

### Intestinal flora genome 16S rDNA sequencing

Total DNA of feces was extracted using a DP328 DNA extraction kit according to the manufacturer’s manual (Tiangen, Beijing, China). The total extracted genomic DNA was qualitatively detected by 1% agarose gel electrophoresis, and the concentration was determined using a Qubit® dsDNA HS Assay Kit. For the 16S rDNA V3 region, an upstream primer 338F and a downstream primer 534R were used for amplification and sequencing on Illumina HiSeq2500 platform (Novogene, Beijing, China).

### 16S rDNA sequence intestinal flora analysis

The OTU was compared using RDP classifer (v 2.2). Greengene database was used for 16S bacteria and archaea genome comparison. Sliver database was used for 18S fungus and UNITE database was used for ITS fungus. The Observed Species, Chao 1 index, Ace index, Shannon index, Simpson index, and Good’s coverage was selected to reflect the Alpha diversity of the samples. PICRUSt was used to perform three-level Kyoto Encyclopedia of Genes and Genomes (KEGG) pathway and abundance analysis based on different numbers of 16S rRNA copy numbers.

### Statistical analyses

The quantitative data were expressed as mean ± standard deviation. The mean of the two groups was compared by student’s t-test. The mean of multiple groups was compared by ONE-WAY ANOVA analysis. The chi-square test was used to compare the rates between groups. Principal co-ordinates analysis was used to analyze the differences in beta diversity among different groups. The dominant intestinal type was analyzed by Multi-dimensional cluster analysis and Principal Component Analysis. All statistical analyses were performed using SPSS v22.0 (SPSS Inc., Chicago, IL, USA), with *p* < 0.05 set as the difference test level.

## Results

### Basic clinical characteristics

A total of 166 patients were enrolled in the study, with 17 in CKD group, 47 in CT group, 49 in HD group, and 53 in PD group. No significant differences in age, gender, and body weight were found among the four groups (*p* > 0.05). Renal function tests, including uric acid, blood urea, nitrogen, and serum creatinine in the healthy control group were significantly lower than those in the other three groups (*p* < 0.001). Metabolic status, including the expression levels of albumin and TC, were significantly higher in CT group (*p* < 0.001), while FBG showed no difference among groups (*p* = 0.29) (Table 1). Primary kidney diseases were altered among three groups of CKD patients, cases of chronic nephritis in CKD, HD, and PD was 4(23.5%), 16 (32.7%) and 21(39.6%), the number of hypertensive nephropathy in CKD, HD, and PD was 10(58.8%), 27(55.1%) and 22 (41.5%) (*p* = 0.002). In inflammatory conditions, CRP and IL-6 were significantly higher in CKD patients (*p* < 0.001) while WBC showed no difference among four groups (*p* = 0.18). CKD patients with PD showed a trend of longer dialysis vintage compared to those with HD (39.85 ± 39.44 month vs. 35.35 ± 32.33 month, *p* = 0.532). Comorbidities including coronary disease and hypertension showed no difference between PD and HD populations (*p* = 0.305 and *p* = 0.327). No significant difference was found on distribution of NYHA classifications (*P* = 0.117). (Table 1).

**Table 1:** Comparison of basic clinical information of patients

	CKD (*N* = 17)	CT(*N* = 47)	HD(*N* = 49)	PD(*N* = 53)	Total	F/χ^2^ value	*p* value
M:F	13:4	23:24	28:21	29:24	93:73	3.91	0.272
Age	57.29 ± 11.13	56.30 ± 10.67	59.53 ± 10.57	57.70 ± 8.52	57.80 ± 10.03	0.85	0.47
Weight (kg)	57.70 ± 13.97	62.49 ± 8.10	58.12 ± 11.63	59.73 ± 11.03	59.83 ± 10.88	1.58	0.197
BUN (mmol/L)	25.86 ± 7.80	4.63 ± 0.93	23.53 ± 7.21	18.63 ± 6.61	16.85 ± 10.00	100.37	< 0.001
Creatinine (umol/L)	1022.94 ± 349.12	67.38 ± 15.53	963.14 ± 286.98	864.09 ± 288.10	684.02 ± 464.44	133.86	< 0.001
UA (umol/L)	452.80 ± 82.88	318.39 ± 63.24	453.63 ± 88.20	431.17 ± 89.58	408.08 ± 99.28	26.99	< 0.001
Albumin (g/L)	35.65 ± 4.05	42.13 ± 3.33	35.06 ± 4.67	32.97 ± 4.35	36.45 ± 5.54	43.55	< 0.001
Hb (g/L)	110.12 ± 14.10	136.72 ± 11.53	105.84 ± 16.59	101.11 ± 24.59	113.51 ± 23.42	36.59	< 0.001
WBC (10^9/L)	7.00 ± 1.96	6.27 ± 1.38	7.35 ± 2.83	7.12 ± 2.99	6.94 ± 2.50	1.67	0.18
CRP	5.29 ± 2.67	2.21 ± 0.99	5.13 ± 2.67	10.26 ± 21.04	5.96 ± 12.36	3.86	0.01
IL-6	6.68 ± 2.98	2.90 ± 2.52	6.61 ± 3.32	12.63 ± 15.30	7.49 ± 9.71	10.02	< 0.001
FBG	4.93 ± 0.66	5.19 ± 0.79	5.13 ± 0.67	5.70 ± 3.13	5.31 ± 1.87	1.24	0.29
HbAc	5.27 ± 0.51	4.91 ± 0.51	5.38 ± 0.66	5.30 ± 0.94	5.21 ± 0.73	3.98	0.009
TC	4.26 ± 4.05	4.85 ± 0.67	4.35 ± 1.00	5.10 ± 1.16	4.72 ± 1.02	6.69	< 0.001
Dialyze number (3/4/5)	–	–	–	5/45/3		–	–
Dialysis vintage (month)	–	–	35.35 ± 32.33	39.85 ± 39.44		0.63	0.532
Primary disease							
Chronic nephritis	4 (23.5)	–	16 (32.7)	21 (39.6)		23.792	0.002
Hypertensive nephropathy	10 (58.8)	–	27 (55.1)	22 (41.5)			
Others	3 (17.6)	–	6 (12.2)	10 (18.9)			
NYHA classification							
I			20 (40.8)	24 (45.3)		4.291	0.117
II			17 (34.7)	24 (45.3)			
III			12 (24.5)	5 (9.4)			
Coronary disease						1.051	0.305
Yes			29 (59.2)	26 (49.1)			
No			20 (40.8)	27 (50.9)			
Hypertension						0.962	0.327
Yes			35 (71.4)	33 (62.3)			
No			14 (28.6)	20 (37.7)			

(CKD: chronic kidney disease; CT: control; HD: hemodialysis; PD: peritoneal dialysis; M: male; F: female; BUN: Blood Urea Nitrogen: UA: Uric Acid: Hb: Hemoglobin: WBC: White Blood Ccll: CRP: C-reactive protein: IL-6: Interleukin-6: FBG: Fasting Blood Glucose: HbAc: Glycated hemoglobin: TC: Total cholesterol)

### Alpha-beta diversity analysis of intestinal flora

**Fig. 1:**
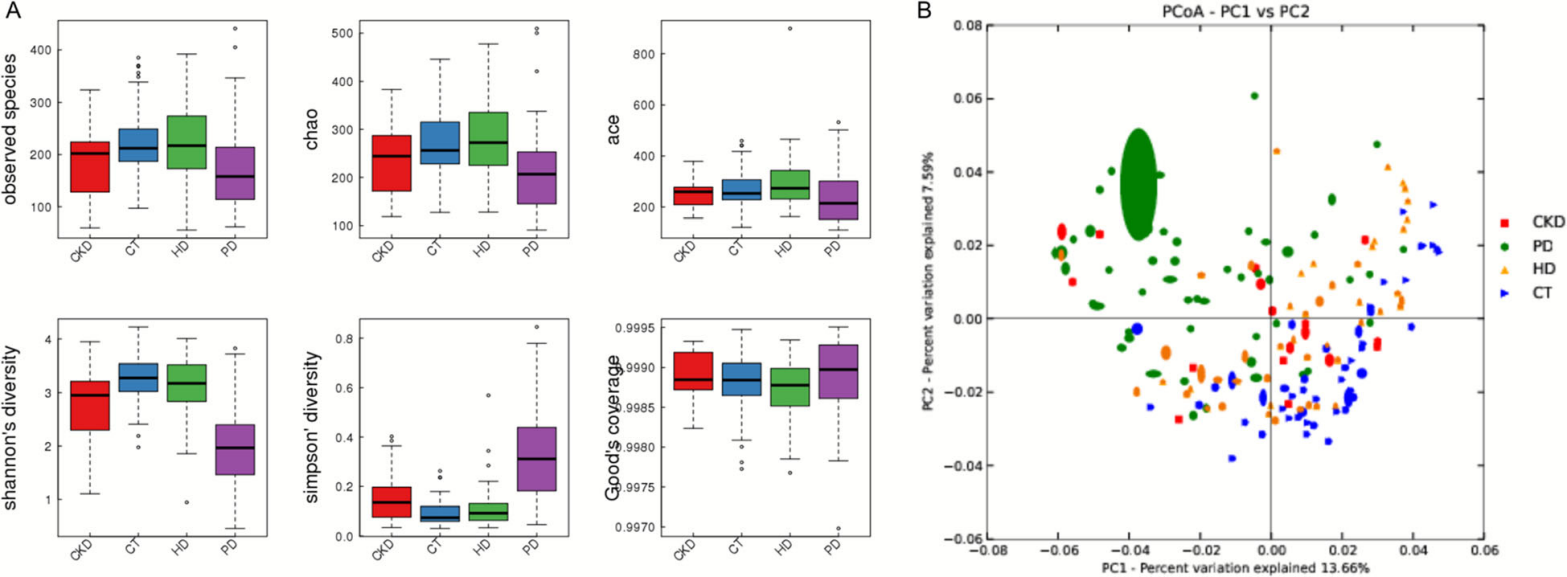
Analysis of alpha and beta diversity of intestinal flora. **a** Intestinal flora structure diversity including Sob index, Chao index, Ace index, Shannon index and Simpson index in PD group was significantly lower than CKD, CT, and HD groups under the same measurement depth. **b** Species diversity distance in PD group was significantly lower than CKD, CT, and HD groups.

**Fig. 2:**
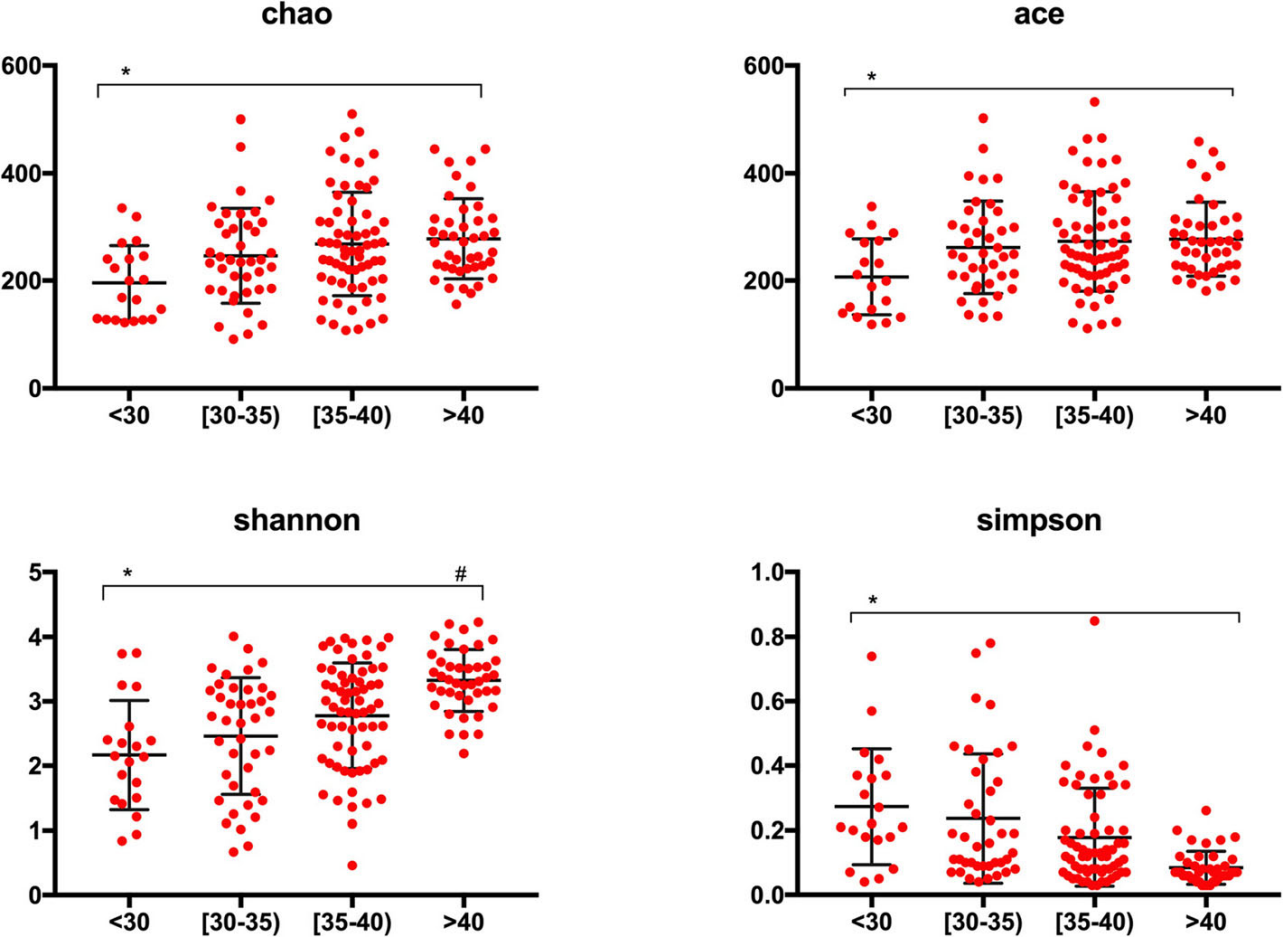
Correlation between Alpha diversity and albumin levels. Intestinal flora alpha diversity including Chao index, Ace index, Shannon index and Simpson index were positively related with albumin levels in 4 grades: < 30 g/L, 30–35 g/L, 35–40 g/L and > 40 g/L.

Alpha and beta diversity analysis showed that the intestinal flora structure diversity (including Sob index, Chao index, Ace index, Shannon index, and Simpson index) and species diversity distance in PD group was significantly lower than CKD, CT, and HD groups under the same measurement depth (*p* < 0.001) (Fig. 1). Further analysis showed that the express of albumin affects the alpha diversity, patients showed with lower albumin level present lower intestinal flora structure diversity and vice visa (Fig. 2).

### Different relative abundance level of bacterial taxa

**Fig. 3:**
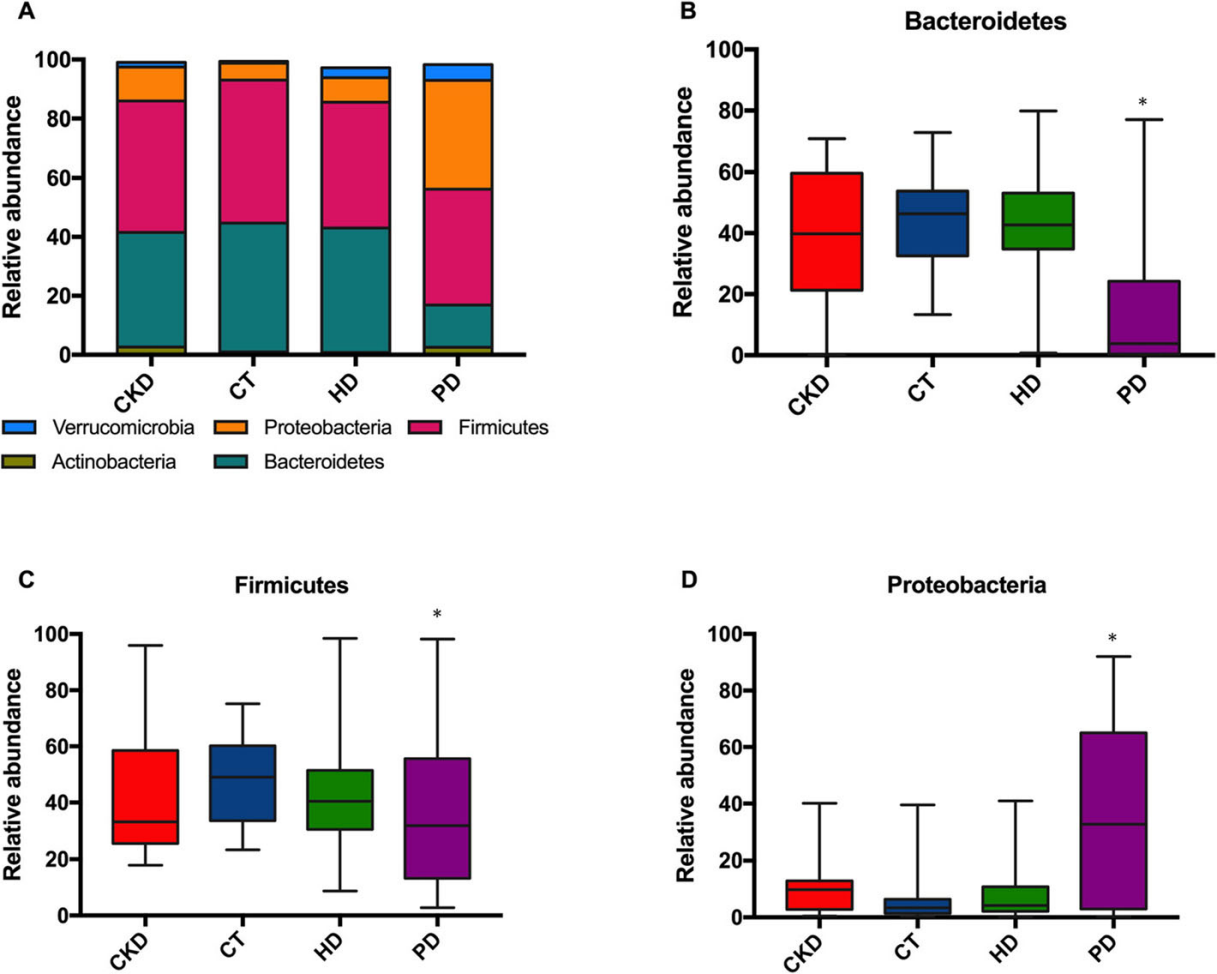
Profiling histogram at the phylum classification level. **a**. Intestinal flora phyla were mostly abundance in Firmicutes, Bacteroidetes, Proteobacteria, Actinobacteria, and Verrucomicrobia. **b**. Bacteroides-dominant gut type was significantly decreased in PD group. **c**. Firmicutes-dominant type was significantly decreased in PD group. **d**. Proteobacteria-dominant gut type was significantly increased in PD group

A total of 20 intestinal flora phyla were detected in 166 fecal samples, namely Firmicutes, Bacteroidetes, Proteobacteria, Actinobacteria, Verrucomicrobia, Fusobacteria, Cyanobacteria, Synergistetes, Tenericumtes, TM7, Chloroflexi, Lentisphaerae, Euryarchaeota, Acidobacteria, Chlamydiae, Spirochaetes, OD1, Elusimicrobia, Parvarchaeota, and unclassified, mostly abundance in Firmicutes, Bacteroidetes, Proteobacteria, Actinobacteria, and Verrucomicrobia (Fig. 3a). All samples were divided into 3 dominant intestinal types including Bacteroides-dominant gut type, Firmicutes-dominant type and Proteobacteria-dominant gut type, which displayed significantly different distributions among the four treatment groups (*p* < 0.01). The relative abundance of bacteria within the phylum Bacteroidetes and Firmicutes were significantly decreased, while relative abundance of the phylum Proteobacteria was increased in PD group (Fig. 3b-d).

### The genus level composition of each group of intestinal flora

**Fig. 4:**
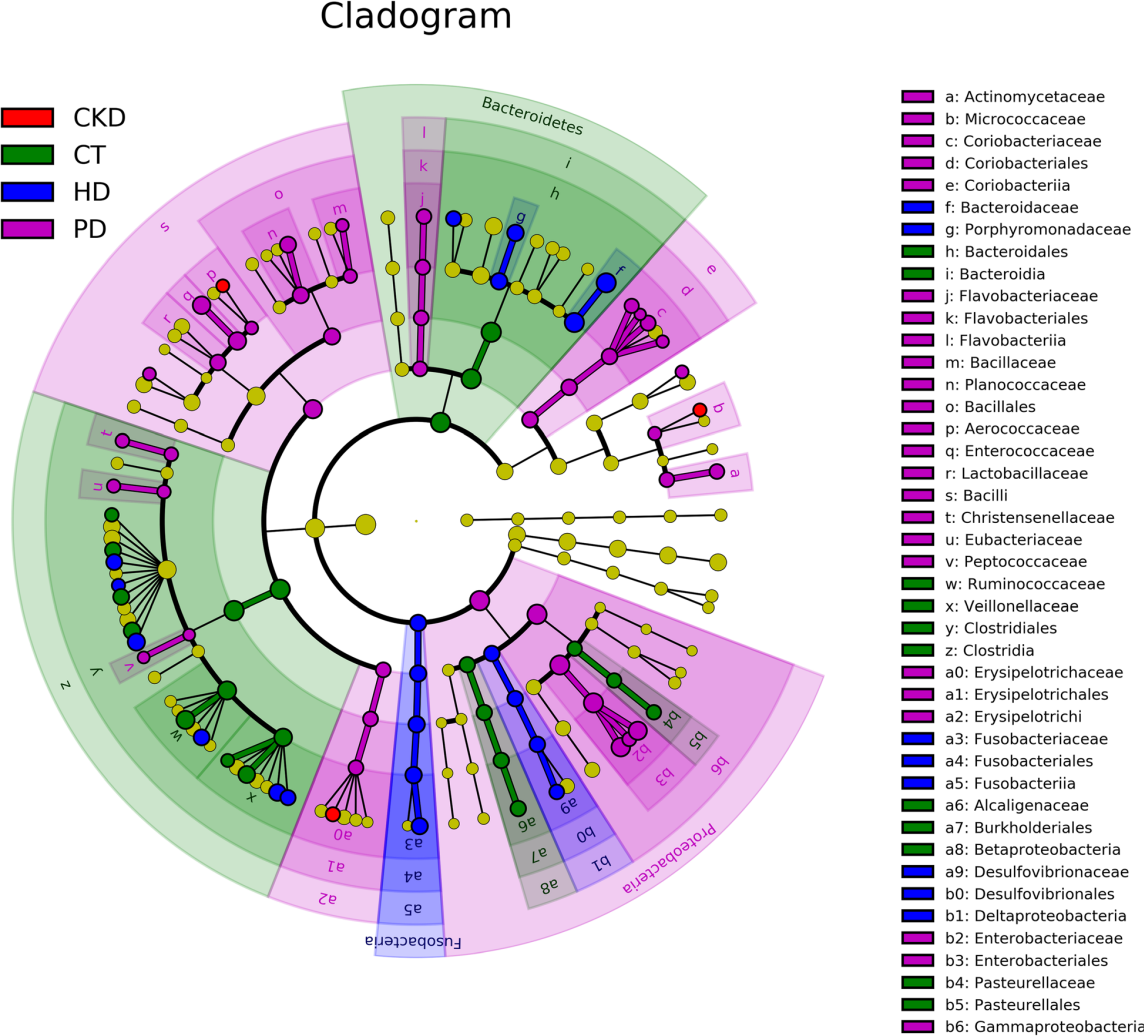
Differences in abundance of bacteria genus between Case group and Control group. Abundance of bacteria genus by LEfSe clustering tree. Different colors indicate different groupings, nodes of different colors represent microbial groups that play an important role in the group represented by the color, one color circle represents a biomarker, and the upper right corner is a biomarker name. Yellow node table Shown are microbial groups that do not play an important role in different groups.

**Fig. 5:**
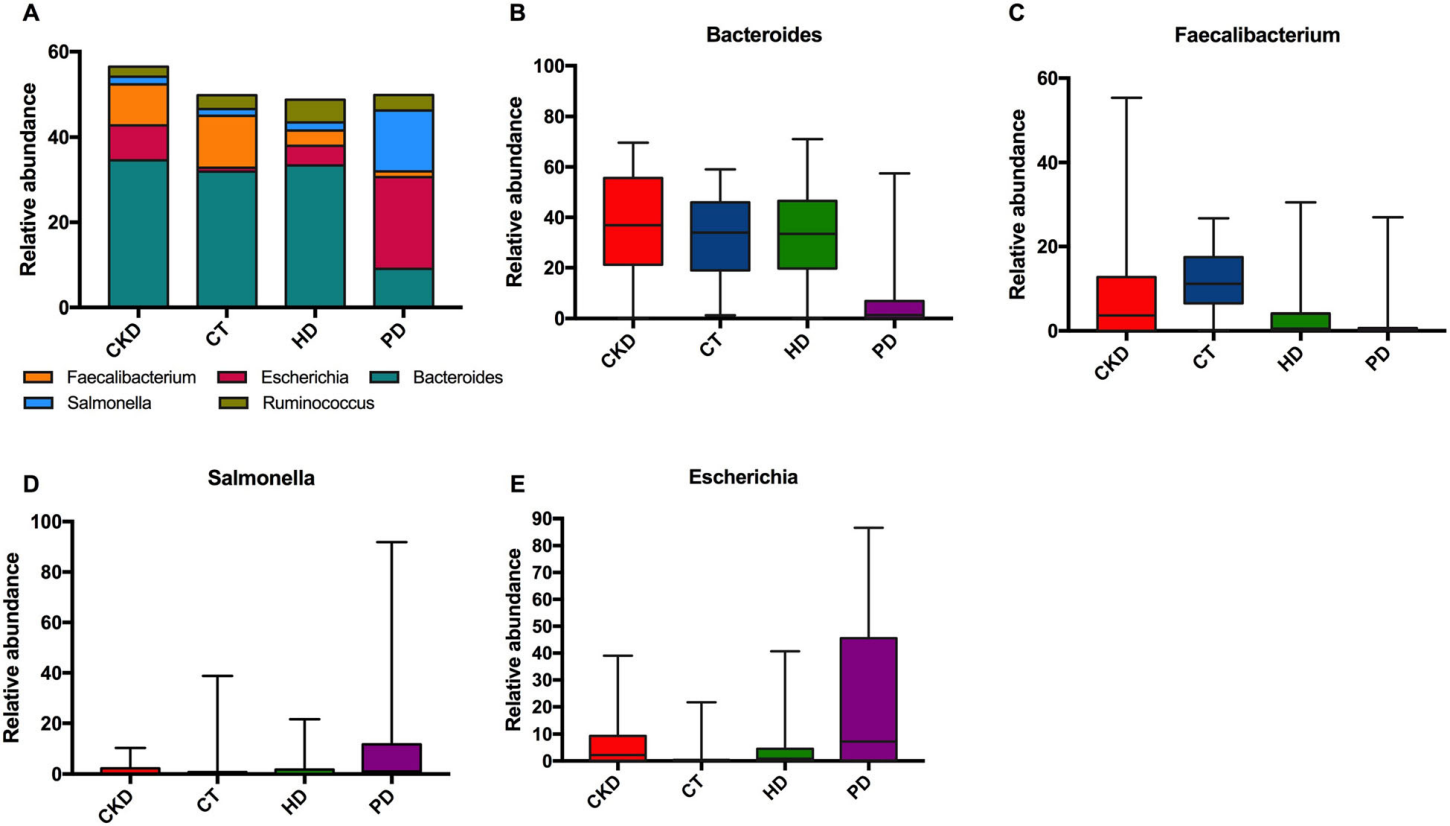
Profiling histogram at the genus classification level. **a**. Bacteroides, Faecalibacterium, Escherichia, Salmonella and Ruminococcus were dominate genus in PD, CKD, CT, and HD groups. **b**. Bacteroides was dominant genus in CT, CKD, and HD groups. **c**. Faecalibacterium was dominant genus in CT, CKD, and HD groups. **d**. Escherichia was dominated genus in PD group. **e**. Salmonella was dominated genus in PD group

Further analyses found 198 genera, the abundance of 86 genera were significantly different (Fig. 3b). 42 genera belong to the Bacteroidetes (7), Proteobacteria (7), Firmicutes (17), and Actinobacteria (11) phyla were significantly different in PD group, 20 genera belonging to Bacteroidetes (5), Proteobacteria (4), Fusobacteria (5), and Firmicutes (6) were significantly different in HD group, 21 genera belonging to Bacteroidetes (3), Proteobacteria (7), and Firmicutes (11) were significantly different in CT group and 3 genera in CKD group belonging to Bacteroidetes (1), Firmicutes (1), Actinobacteria (1) were significantly different (Fig. 4). 4 dominant genera among four groups were: *Bacteroides, Faecalibacterium, Escherichia* and *Salmonella*. *Bacteroides* and *Faecalibacterium* were dominant genus in CT, CKD, and HD groups, while *Escherichia* and *Salmonella* was dominated genus in PD group (Fig. 5).

### Relative abundance of indole and p-cresol producing taxa in family level

**Fig. 6:**
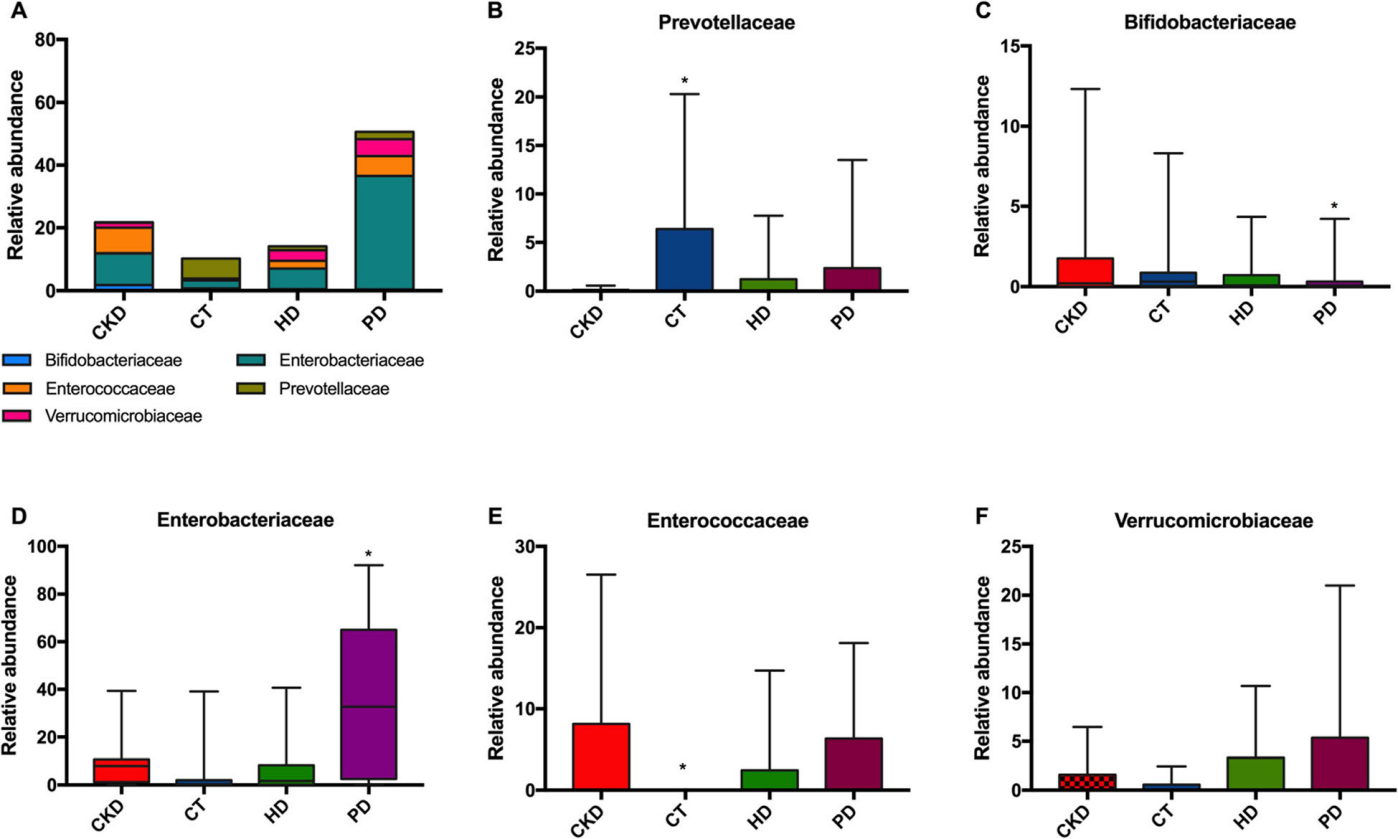
Profiling histogram at the family classification level. **a**. Bifidobacteriaceae, Prevotellaceae, Enterobacteriaceae, Enterococcaceae, and Verrucomicroblaceae were dominate family in PD, CKD, CT, and HD groups. **b**. Prevotellaceae was significantly decreased in patients with PD. **c**. Bifidobacteriaceae was significantly decreased in patients with PD. **d**. Enterobacteriaceae was significantly increased in patients with PD. **e**. Enterococcaceae was significantly increased in patients with PD. **f**. A trend of increased level was found in the relative abundance of Verrucomicroblaceae

*Bifidobacteriaceae* and *Prevotellaceae* was significantly decreased, while *Enterobacteriaceae*, *Enterococcaceae* were significantly increased in patients with PD compared with the other groups. A trend of increased level was found in the relative abundance of *Verrucomicroblaceae* (Fig. 6).

### Analysis of intestinal microbial function

**Fig. 7:**
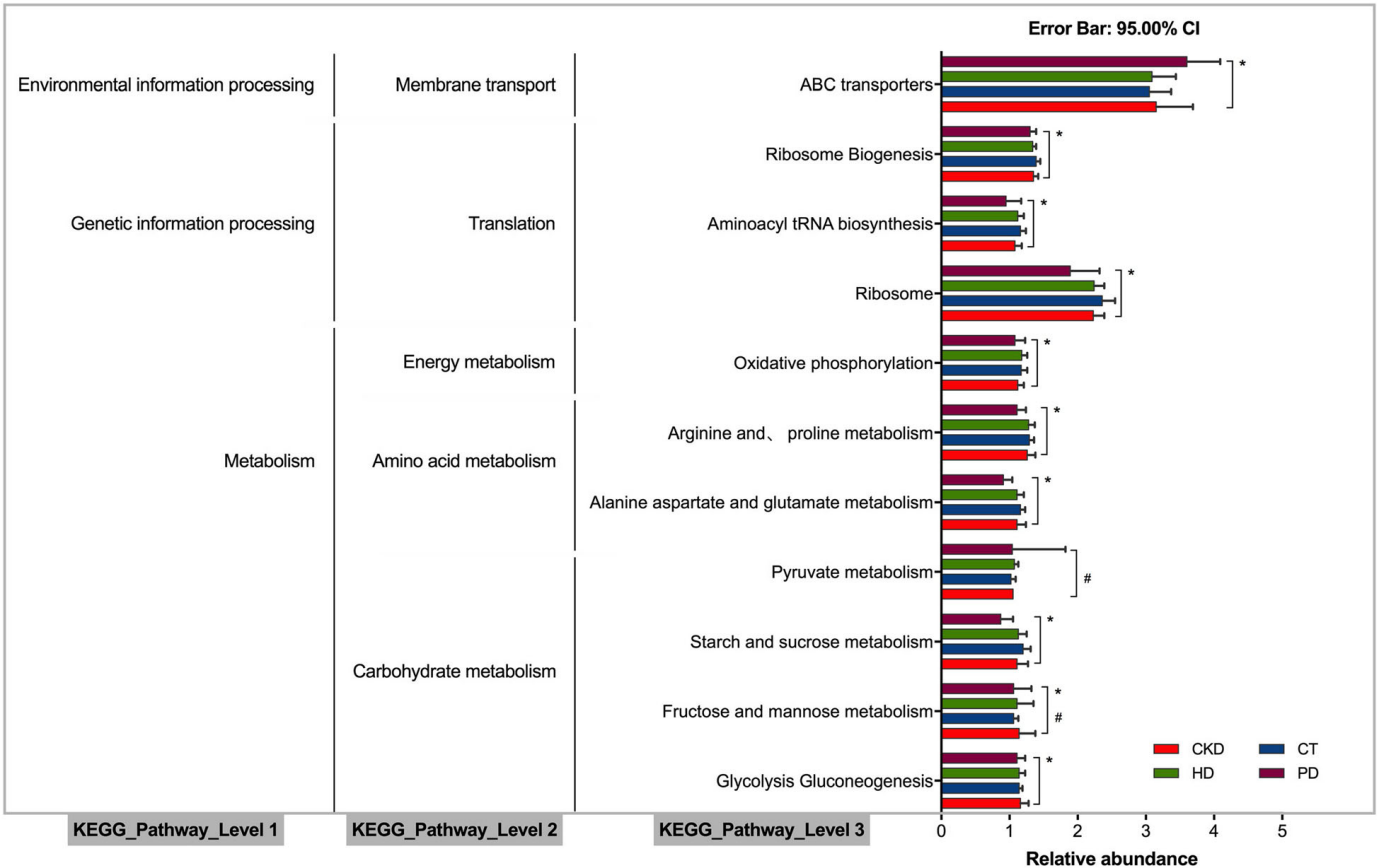
KEGG pathway of abundance changed intestinal flora. Three levels of KEGG enrichment pathways in CT, CKD, HD and PD group. High-abundance bacteria KEGG level 1 pathways were significantly enriched in metabolism, genetic information processing, and environmental information processing. The KEGG level 2 pathways were significantly enriched in carbohydrate metabolism, amino acid metabolism, energy metabolism, translation, and membrane transport. The KEGG level 3 pathways were significantly enriched in starch and sucrose metabolism, alanine aspartate and glutamate metabolism, arginine and proline metabolism, oxidative phosphorylation, ribosome, aminoacyl tRNA biosynthesis, and ABC transporters.

High-abundance bacteria KEGG level 1 pathways in the four groups were significantly enriched in metabolism, genetic information processing, and environmental information processing. The following KEGG level 2 pathways were significantly enriched in carbohydrate metabolism, amino acid metabolism, energy metabolism, translation, and membrane transport. Number of differential expressed genes in KEGG enrichment pathways: starch and sucrose metabolism, alanine aspartate and glutamate metabolism, arginine and proline metabolism, oxidative phosphorylation, ribosome, aminoacyl tRNA biosynthesis, and ABC transporters were significantly different in PD group compared with CT, CKD, and HD groups (Fig. 7 & Additional file 1: Table S1).

## Discussion

Gut microbiota affect physiological functions in CKD patients by modulating genes involved in host immunity, cell proliferation, and metabolism [17–19]. The pattern of renal replacement therapy also appears to influence gut microbiota [20]. Our study showed that decreased diversity and altered communities of intestinal flora in PD patients, in which microbial diversity was positive correlated with the albumin level. A total of 20 intestinal flora phyla were detected in 166 fecal samples, divided into 3 dominant intestinal types including Bacteroides-dominant gut type, Firmicutes-dominant type and Proteobacteria-dominant gut type. Further analyses found 198 genera, the abundance of 86 genera were significantly different. Butyrate-producing taxa as *Faecalibacterium* in genera level and *Bifidobacteriaceae* and *Prevotellaceae* in family level were dominant genus in CT, CKD, and HD groups, while Urease containing-, indole- and p-cresol-forming taxa as Escherichia in genera and *Enterobacteriaceae, Enterococcaceae* in family level was dominated genus in PD group. Number of differential expressed genes in KEGG enrichment pathways were significantly different in PD group in carbohydrate metabolism, amino acid metabolism, energy metabolism, translation, and membrane transport.

Qualitative and quantitative changes in host microbiome profile and disruption in gut barrier resulting in gut dysbiosis was commonly seen among CKD patients [21]. A Chinese study observed no significant differences in intestinal flora diversity between CKD patients and healthy control groups, suggesting that bacterial diversity was not seriously damaged in this population [14]. Microbiota dysbiosis, which was differed between modes of dialysis, was considered a main risk factor in promoting chronic systemic inflammation in CKD patients [22]. Besides dialysis modes, age and dialysis vintage also contributed to the microbiome diversity [20]. Another Chinese study found that probiotic bacteria was less frequently detected in PD patients, which may impair host intestinal barrier and increase the risk of enteric organism invasion [23]. Diabetic patients could develop impaired renal function and induce diabetic associated cardiovascular disease [24, 25]. These diabetic cardiomyopathy patients may have abnormal bacterial metabolism [26]. Our study has further confirmed that different dialysis modes were critical contributors to microbiota alterations considered that diabetic patients have been excluded and no cardiac dysfunction have been found in our patients.

High-throughput sequencing in our study found that the intestinal flora diversity of PD patients was lower than that of HD and non-dialysis CKD patients, suggested that intestinal flora was seriously damaged by PD as a renal replacement therapy. Other investigation further revealed that alpha diversity was closely related to the patient’s inflammatory condition [14, 20]. Significant relationship between diversity and inflammatory factors was not found in our study, however, we revealed that patients who have higher albumin level showed with more abundance intestinal flora. This result suggested that people with better alpha diversity of the flora could have better nutrition. Improve the diversity of bacteria could be an effective way to improve the malnutrition status of dialysis patients.

Colonic bacteria ferment indigestible carbohydrates and proteins and form short chain fatty acids (SCFAs), so that distal guts can absorb more rapidly. SCFAs were also used as cross-feeding nutrients for microorganisms, which were unable to digest macromolecules [27, 28] SCFAs can bind to G-protein-coupled receptors GPR41 and GPR43 for regulating inflammation in adipose tissue, intestinal cells, and immune cells [29, 30]. A large number of homologous peptides between human and intestinal bacteria have an autoimmunogenicity effect by binding to HLA-II alleles. Most of the bacteria containing autoimmune peptides were belonged to either the phylum Firmicutes or Proteobacteria [31]. Our study showed the abundance of Proteobacteria was higher while Bacteroidetes and Firmicutes were lower in the PD group. This result suggested that the decreased Proteobacteria in patients undergoing PD might aggravate the disease situation. Further analyses on genus level revealed that compared with the other three groups, *Bacteroides, Escherichia* and *Faecalibacterium* was the dominant intestinal type in the PD group. *Escherichia* produce indoles which mainly affect the cardiovascular system and kidney functions and *Faecalibacterium* is one of the most abundant human fecal bacterial populations that produce butyrate, suggesting that PD treatment may increase toxins released in CKD patients [32, 33]. With the accumulation of uremic toxins and activation of inflammatory reactions in CKD patients, there is a decrease in qualitative and quantitative properties of probiotics, which in turn promotes CKD progress [34, 35]. Changed family population of *Bifidobacteriaceae, Prevotellaceae* and *Lactobacillaceae* can result in reduced butyrate level and lead to insufficient SCFAs, while families possessed Tryptophanase, indole and p-cresol-forming enzymes including *Clostridiacease, Enterobacteriaceae* and *Verrucomicrobiaceae* [36]. In our study, decreased SCFA secretion induced by decreased *Bifidobacteriaceae* and *Prevotellaceae*, increased toxins secretion induced by *Enterobacteriaceae* and *Enterococcaceae* indicated that changed population of intestinal flora may have an adverse effect on CKD patients with PD.

Gut microbiota can also affect immune system stimulation, intestinal epithelial homeostasis, vitamins B and K synthesis, gastrointestinal motility and function enhancement, nutrients absorption, drugs metabolism and SCFAs and polyamines production [4]. In our study, similar CKD and HD intestinal flora was correlated with similar expression of CRP and IL-6. However, significantly decreased diversity and altered communities of intestinal flora between PD and HD was found correlated with excessive expression of CRP and IL-6. Butyrate-producing taxa as Faecalibacterium in genera level and Bifidobacteriaceae and Prevotellaceae in family level were dominant genus in CT, CKD, and HD groups, while urease containing-, indole- and p-cresol-forming taxa as Escherichia in genera and Enterobacteriaceae, Enterococcaceae in family level was dominated genus in PD group. Our result indicated that beneficial and harmful bacteria was imbalanced in PD patients, which was more likely to induce inflammatory response of ESRD patients. CRP and IL-6 as inflammatory markers need further analysis. In this study, functional analysis of the microbiome with altered expression in CKD patients under different therapeutic states suggests that changes in the abundance of certain microbial species in plays an important role in affecting metabolic functionality and inflammatory response. Notably, a regulatory effect on bacterial chemotaxis was found in level 3 KEGG analysis. Bacteria moved under the control of a complex signal transduction system, moving toward the production of beneficial chemicals or away from unfavorable chemicals. The signal of bacterial chemotaxis may be the mechanism of escaping the antibacterial effect of antibiotics and aggravating the disease, although future investigation is required to confirm this.

The present study has certain limitations. First, this was a single-center research, the generalizability of altered microbiota in CKD patients with different hemodialysis therapy is limited. Second, the lack of animal model experiments to validate the effect of improved microbial diversity to patient’s malnutrition status was also a limitation in our study. Further CKD animal models were needed in verify to verify the possible role of intestinal flora in recovery of CKD patients. In spite of these limitations, our results provide information regarding the altered microbial diversity and communities in Chinese CKD patients with different hemodialysis therapy and suggest that we should raise attention on intestinal flora of CKD patients with PD therapy. The influence of different primary renal diseases on microbiota alteration needs further investigation.

## Conclusion

In conclusion, peritoneal dialysis therapy could result in reduced diversity and altered microbial communities, with reduced probiotic butyrate-producing taxa and increased urease containing-, indole- and p-cresol-forming taxa. The disordered intestinal flora can seriously affect the nutrition level in CKD patients with PD therapy.

## Supplementary information

**Additional file 1 Table S1.** KEGG level 3 pathways of abundance changed intestinal flora.

## Data Availability

All data generated or analyzed during this study are included in this published article.

## Abbreviations

CKD: Chronic kidney disease
CT: Control
HD: Hemodialysis
KEGG: Kyoto Encyclopedia of Genes and Genomes
PD: Peritoneal dialysis
SCFAs: Short-chain fatty acids
TMAO: Trimethylamine N-oxide
