# Supplementary material for: High-throughput sequencing analysis of intestinal flora changes in ESRD and CKD patients
Source: BMC Nephrol. 2020 Jan 13;21:12. doi: 10.1186/s12882-019-1668-4 (PMC6958730; doi:10.1186/s12882-019-1668-4)
Supplement: Supplementary file 1 — Additional file 1 Table S1. KEGG level 3 pathways of abundance changed intestinal flora. [file 12882_2019_1668_MOESM1_ESM.docx]

Table S1 Differential KEGG enrichment pathways

| KEGG pathway | CKD | CT | HD | PD | F value | P value |
| --- | --- | --- | --- | --- | --- | --- |
| Amino_Acid_Metabolism | 9.58 | 9.89 | 9.78 | 8.87 | 35.829 | <0.001 |
| Biosynthesis_of_Other_Secondary_Metabolites | 0.97 | 1.02 | 1.00 | 0.78 | 45.714 | <0.001 |
| Carbohydrate_Metabolism | 11.25 | 11.04 | 11.12 | 10.68 | 7.664 | <0.001 |
| Cell_Growth_and_Death | 0.50 | 0.53 | 0.51 | 0.42 | 28.834 | <0.001 |
| Cell_Motility | 1.39 | 1.83 | 1.63 | 2.11 | 7.421 | <0.001 |
| Cellular_Processes_and_Signaling | 4.30 | 4.27 | 4.31 | 4.70 | 8.819 | <0.001 |
| Energy_Metabolism | 5.68 | 5.92 | 5.84 | 5.30 | 28.945 | <0.001 |
| Enzyme_Families | 2.14 | 2.19 | 2.15 | 2.12 | 5.985 | 0.001 |
| Folding_Sorting_and_Degradation | 2.50 | 2.57 | 2.51 | 2.35 | 16.789 | <0.001 |
| Genetic_Information_Processing | 2.71 | 2.64 | 2.70 | 2.87 | 11.172 | <0.001 |
| Glycan_Biosynthesis_and_Metabolism | 2.58 | 2.48 | 2.56 | 2.40 | 1.159 | 0.327 |
| Infectious_Diseases | 0.42 | 0.38 | 0.40 | 0.53 | 37.253 | <0.001 |
| Lipid_Metabolism | 2.89 | 2.81 | 2.91 | 2.91 | 2.617 | 0.053 |
| Membrane_Transport | 12.19 | 11.36 | 11.70 | 13.93 | 23.365 | <0.001 |
| Metabolism | 2.70 | 2.46 | 2.64 | 3.08 | 41.363 | <0.001 |
| Metabolism_of_Cofactors_and_Vitamins | 4.30 | 4.53 | 4.38 | 4.09 | 22.036 | <0.001 |
| Metabolism_of_Other_Amino_Acids | 1.53 | 1.51 | 1.51 | 1.52 | 0.472 | 0.703 |
| Metabolism_of_Terpenoids_and_Polyketides | 1.62 | 1.60 | 1.63 | 1.56 | 3.486 | 0.017 |
| Nucleotide_Metabolism | 3.98 | 4.03 | 3.93 | 3.62 | 16.567 | <0.001 |
| Poorly_Characterized | 5.07 | 4.86 | 4.97 | 5.26 | 20.534 | <0.001 |
| Replication_and_Repair | 8.57 | 8.97 | 8.69 | 7.71 | 32.819 | <0.001 |
| Signal_Transduction | 1.54 | 1.45 | 1.52 | 2.05 | 29.904 | <0.001 |
| Transcription | 2.89 | 2.86 | 2.89 | 3.05 | 12.223 | <0.001 |
| Translation | 5.36 | 5.64 | 5.42 | 4.76 | 25.231 | <0.001 |
| Transport_and_Catabolism | 0.36 | 0.34 | 0.34 | 0.24 | 16.560 | <0.001 |
| Xenobiotics_Biodegradation_and_Metabolism | 1.67 | 1.52 | 1.67 | 1.91 | 38.359 | <0.001 |
| Others | 1.31 | 1.30 | 1.29 | 1.17 | 10.080 | <0.001 |
